# Dairy milk: There are alternatives but no equivalents

**DOI:** 10.1002/fsn3.4301

**Published:** 2024-07-19

**Authors:** Emma L. Beckett, Tim Cassettari, Carlene Starck, Flávia Fayet‐Moore

**Affiliations:** ^1^ FOODiQ Global Sydney New South Wales Australia; ^2^ School of Environmental and Life Sciences The University of Newcastle Callaghan New South Wales Australia; ^3^ School of Health Sciences The University of New South Wales Sydney New South Wales Australia

**Keywords:** dairy, dairy alternatives, environment, health, milk, plant based, retail

## Abstract

Dairy milk is a core food in many food‐based guides to healthy eating. However, plant‐based milk alternatives are becoming increasingly available as substitutes. While these products serve a subset of the population unable or unwilling to consume milk, plant‐based milk alternatives can be perceived by consumers as direct equivalents, or even more healthful alternatives to dairy milk. This commentary addresses the significant differences in nutrient content that may have implications for the intake of key nutrients in the case of direct substitutions. Furthermore, while there is a significant body of knowledge demonstrating the significant health benefits associated with dairy milk consumption and a small number of potentially negative associations, there is a paucity of data on the health benefits of plant‐based milk alternatives directly. A “health halo” may exist based on matching individual nutrients through fortification, lower energy levels, and the health properties of the unprocessed raw characterizing ingredients of plant‐based milk alternatives. This may mislead consumers regarding healthfulness. Similarly, environmental attributes based on volumes of production, without considering contribution to nutrients, may also skew consumer perception. Positioning of plant‐based milk alternatives in food‐based dietary guidelines, marketing, and personal recommendations should acknowledge the differences in nutritional, bioactive, and health properties between plant‐based milk alternatives and dairy milk to ensure appropriate adaptations are made to account for shortfalls in nutrients.

## Graphical abstract

Dairy milk is highly nutritious and yet plant‐based milk alternatives are becoming increasingly available as substitutes, serving those who are unable or unwilling to consume dairy milk. However, positioning these alternatives as equivalents when they are different in their profiles of nutrients, bioactives, matrix features, processing, and health outcomes evidence could have consequences for population‐level nutrient intakes, and this should be considered in public recommendations.

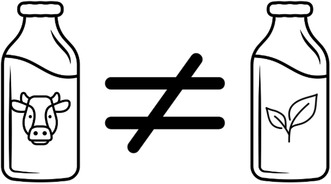

## 1 INTRODUCTION

### 1.1 The growth of plant‐based milk alternatives

Dairy milk, consumed as beverages or with foods such as cereals, has long been a widely consumed core and staple food in the Western diet. While no single food or beverage is essential, milk features in many food‐based dietary guidelines (Comerford et al., 2021) and makes significant contributions to nutrition globally (Smith, Dave, et al., 2022). However, the interest in and availability of plant‐based milk alternatives, typically made from grains, seeds, and legumes, have significantly expanded in recent years. Ethical, environmental, and health‐related perceptions (including, but not limited to, lactose intolerance, allergies, weight loss, and lowering fat intake) are typically cited as motivating factors by consumers for substitution (Haas et al., 2019; McCarthy et al., 2017; Mylan et al., 2019; Slade, 2023). Plant‐based milk alternatives are one of the fastest‐growing plant‐based food product sectors, and growth has been forecast to continue by approximately 15% per annum through 2030 (https://www.databridgemarketresearch.com/reports/global‐plant‐based‐milk‐market). A 2020 study found 115 unique plant‐based milk alternative products available in major Australian supermarkets (Zhang et al., 2020), and a 2019 study in the United Kingdom found 82 (Sumner & Burbridge, 2020). Subsequently, demand for dairy milk has declined (Slade, 2023). Recent data from the USA found that for every 1‐gallon increase in sales of plant‐based milk alternatives, there was an associated 0.42–0.60 gallon reduction in dairy milk sales (Slade, 2023).

### 1.2 Purpose and consumer perceptions

Plant‐based milk alternatives are often marketed as direct substitutes for dairy milk (Sethi et al., 2016), with formulations to produce similar color, texture, and mouthfeel for matching culinary purposes. However, plant‐based milk alternatives, which are primarily the aqueous or reconstituted extracts of plant ingredients, including nuts, legumes, grains, and others, do not fit the biological definition of milk (the mammary secretion of milking animals) and do not meet the definitions used in many food standards and codes, including in the Codex Alimentarius (FAO, 2024). The use of the term “milk” (e.g., soy milk) to describe plant‐based milk alternatives is allowed in some countries (i.e., Australia) but it is not allowed in many jurisdictions, where “drink” or “beverage” (e.g., soy drink or soy beverage) is used to describe plant‐based milk alternatives. Concerns have also been raised with this terminology as milk alternatives may be misleading to consumers as it implies it is equivalent to dairy milk, particularly in the context where plant‐based products may not be interchangeable for consumption by infants or young children in place of dairy milk (Siddiqui et al., 2023). A 2018 survey of adults in the USA reported that while consumers feel that descriptors are sufficient to distinguish plant‐based milk alternatives from dairy milk (Baptista & Schifferstein, 2023), over half said that they believe that plant‐based products were labeled using the word “milk” because their nutritional value is equivalent (IPSOS, 2018).

There is potential that the design of these products for functional and sensory (Pingali et al., 2023) equivalency to dairy milk, and the inclusion of references to plant‐based alternatives alongside dairy in many food‐based guides to healthy eating may inadvertently also imply nutritional and health equivalency (Siddiqui et al., 2023). This may explain why plant‐based milk alternatives are viewed by consumers as more healthful than dairy milk (McCarthy et al., 2017), as the term plant based may be seen to apply equivalent health benefits to that from whole plant foods and beverages (Pingali et al., 2023). Therefore, it is necessary to directly compare and discuss not only the nutritional composition of milk and its plant‐based alternatives but also known health benefits, their places in food‐based healthy eating guidelines, and environmental impact to help guide policy and practice surrounding these “substitute” products.

## 2 NUTRITIONAL COMPOSITION

Dairy milk is featured as a core food in many food‐based healthy eating guides as it is nutritionally dense, and a source of multiple nutrients considered priority or shortfall nutrients (Comerford et al., 2021). Plant‐based milk alternatives can be processed and designed to mimic the nutritional composition of dairy milk, but their composition is not equivalent (Table 1).

**TABLE 1:** Nutritional overview of dairy milk compared to plant‐based milk alternatives.

Feature	Dairy milk	Plant‐based milk alternatives
Sugars	Sugars in unflavored milk are not free sugars, as they are naturally occurring and bound in the milk matrix (Swan et al., 2018) Sugars in milk are mostly lactose (Walther et al., 2022)	Sugars in plant‐based milk alternatives are free sugars (Aydar et al., 2020), even if unsweetened May contain added sugars Sugars in plant‐based milk alternatives are mostly glucose (Walther et al., 2022)
Protein	Cow's milk is a high‐quality protein source with a high provision of essential amino acids and high digestibility (Aydar et al., 2020)	Other than soy milk, plant‐based milk alternatives have lower total protein content (unless fortified with protein) and fewer essential amino acids (Aydar et al., 2020; Drewnowski et al., 2021; Walther et al., 2022)
Fats	0.15% (skim) – 3.5% (whole milk) Mostly saturated fats (~70%) (Djordjevic et al., 2019; Pereira, 2014)	1–3.2% fat Typically mostly unsaturated (Walther et al., 2022), with saturated fats higher in some (e.g., coconut milk; Bharti et al., 2021)
Calcium	Matrix effects enhance bioavailability (Melse‐Boonstra, 2020; Shkembi & Huppertz, 2021) Remains in suspension (Soerensen et al., 2014)	Fortified in less bioavailable forms compared to dairy milk (Aydar et al., 2020) Precipitates and drops out of suspension requiring shaking before consumption (Aydar et al., 2020; Walther et al., 2022)
Other micronutrients	Good source of calcium, protein, iodine, vitamin A, vitamin D, vitamin B_2_, vitamin B_12_, and zinc (NHMRC, 2013; Pereira, 2014)	Fortified plant‐based milk alternatives provide comparable levels of vitamin B_12_, B_2_, and vitamin A No plant‐based milk alternative provides comparable zinc, B_5_, or choline (Chalupa‐Krebzdak et al., 2018; Drewnowski et al., 2021) A limited number of plant‐based alternatives are fortified with iodine (Nicol et al., 2023)

### 2.1 Macronutrients

#### 2.1.1 Energy

The macronutrients supplied by dairy milk have been shown to play a significant role in supplying energy and reducing global hunger (FAO, 2020.). However, plant‐based milk alternatives are often lower in energy content, which is a potential motivating factor for selection when addressing energy balance is a consideration (Haas et al., 2019).

#### 2.1.2 Protein

Dairy milk is a complete source of protein (providing all essential amino acids in sufficient levels). Soy milk is the only plant‐based milk alternative that reflects this protein content and quality, with all other plant‐based milk alternatives being incomplete, as well as being notably lower in protein (Antunes et al., 2022; Smith, Dave, et al., 2022). In an Australian sample protein content in dairy milk ranged from 3.2 to 4.7 g/100 mL, while plant‐based milk alternatives ranged from 0 to 4.2 g/100 mL, with the legume‐based products being the highest, and the nuts and seeds products being the lowest (Zhang et al., 2020). Globally, dairy milk contributes more than 10% of the total protein consumed (Smith, Fletcher et al., 2022). Observational and modeling studies across various age groups and geographic populations have shown that those who include dairy milk in their diet are more likely to meet recommended intakes for protein than those who do not (Cifelli et al., 2016; Fayet‐Moore et al., 2013; Parker et al., 2012; Rangan et al., 2012; Saito et al., 2019). This is unlikely to be of concern for the majority of the population with mixed and balanced diets. However, there may be negative consequences for specific populations, such as older men (Lawrence et al., 2023) and children (Zhang et al., 2020). As such, plant‐based milk alternatives with lower levels of protein than milk are not recommended as complete replacements for children under 5 years of age (Zhang et al., 2020).

#### 2.1.3 Carbohydrates

The presence of lactose as a primary sugar is a reason typically cited for avoidance of dairy milk (Haas et al., 2019). However, complete avoidance is not typically required as complete congenital intolerance is rare (Misselwitz et al., 2019). Furthermore, lactose‐free milk is easily produced via enzymatic digestion. While plant‐based milk alternatives are naturally lactose free, all sugars in plant‐based milk alternatives are free sugars, regardless of whether or not they are added sugars (Sumner & Burbridge, 2020). Therefore, plant‐based milk alternatives have higher cariogenic potential with free sugars fermentable by oral bacteria. Intake of free sugars below 10% of total energy per day is recommended by the World Health Organization (strong recommendation), or less than 5% (conditional recommendation) (WHO, 2015). One standard serve of plant‐based milk alternatives (250 mL) could substantially contribute to this intake, particularly the legume and grain‐based products containing up to 16.25 g of free sugars (Zhang et al., 2020). UK dietary modeling (Clegg et al., 2021) found that plant‐based milk alternatives contributed a significantly greater proportion of the daily intake of free sugars than dairy milk. Dairy milk is a moderate glycemic index (GI) beverage, while plant‐based milk alternatives can be moderate GI or high GI, such as rice milk (Antunes et al., 2022).

#### 2.1.4 Fats

Plant‐based milk alternatives are often lower in fats, which may be a motivating factor for selection and consumption (Haas et al., 2019). However, total fats are highly variable in both dairy milk and plant‐based milk alternatives. This variability is similar across all categories, with levels ranging from 0 to ~3.5 g/100 mL in dairy milk, and grain‐, nut/seed‐, legume‐ and coconut‐based milk alternatives (Zhang et al., 2020). As the characterizing plant ingredients are low in fat, vegetable oils are a common additive to assist with mimicking the creamy mouthfeel of dairy milk. As such, plant‐based milk alternatives are typically higher in mono‐ and poly‐unsaturated fats and lower in saturated fats compared to dairy milk, and contain phytosterols, which may have benefits for managing cholesterol levels (Eslami & Shidfar, 2019; Shin et al., 2003).

### 2.2 Micronutrients

Dairy milk is a good source of vitamin A, iodine, zinc, calcium, and potassium. Vitamin A, iodine, and zinc are among the most common nutrient deficiencies globally, particularly in low‐ and middle‐income regions, and calcium and potassium are of significant public health concern in industrialized nations (Comerford et al., 2021; Starck et al., 2024). A diet low in calcium is a significant contributor to the global burden of disease (Afshin et al., 2019). Dairy milk is the leading contributor to global calcium and vitamin B_2_ availability and is among the top five contributors for 21 additional nutrients (Smith, Fletcher et al., 2022), including accounting for 49% of calcium, 24% of vitamin B_2_, and >10% of vitamins A, B_5_, and B_12_, phosphorus, and potassium globally (Smith, Fletcher et al., 2022).

Modeling the nutritional impacts of replacing dairy milk with plant‐based milk alternatives is highly dependent on the plant source and fortification practices. Observational and modeling studies across various age groups and geographic populations have shown that dairy milk intake is a marker of dietary quality, with consumers more likely to meet intake recommendations for multiple essential micronutrients. The use of plant‐based milk alternatives as a replacement for dairy milk could reduce intakes of protein, calcium, vitamin A, vitamin B_12_, B_2_, B_6_, iodine, *n*‐3 long‐chain fatty acids, and zinc (Cifelli et al., 2016; Fayet‐Moore et al., 2013; Lawrence et al., 2023; Parker et al., 2012; Rangan et al., 2012; Saito et al., 2019; Zhang et al., 2020), with significant impacts on the ability of consumers to meet intake recommendations for protein, zinc, and calcium (Zhang et al., 2020).

Dietary modeling in the United Kingdom (Clegg et al., 2021) found that plant‐based milk alternatives contributed significantly lower proportions of many micronutrients, including vitamins B_2_, B_12_, and iodine, in both children and adults, when replacing dairy milk in the diet. Similarly, recent modeling in Australia showed that if dairy milk was replaced with plant‐based alternatives in the diet, intakes for vitamin B_12_, vitamin B_2_, iodine, niacin, calcium, potassium, phosphorus, and zinc would be adversely impacted (Lawrence et al., 2023; Table 1). In a 2020 cross‐sectional survey of plant‐based milk alternatives in Australia, only 50% were fortified with calcium, one‐third contained a calcium level similar to dairy milk, and all plant‐based milk alternatives were a poor source of vitamin B_12_ (Zhang et al., 2020). In contrast, surveys suggest that consumers believe that both dairy and soy milk are good sources of calcium (Bus & Worsley, 2003). In the United States, over 60% of adults surveyed believed that plant‐based milk alternatives (made from almond, soy, or coconut) had the same key nutrients, or even more, compared to dairy milk (Schiano et al., 2022). As such, the major role that dairy milk plays in nutrient provision means that alternatives should not just be functional equivalents but also nutritional equivalents. Otherwise, significant changes in dietary patterns and public health recommendations would be needed to prevent the unintended exacerbation of existing nutrient shortfalls (Starck et al., 2024), including revisions in food‐based dietary guidelines that recommend dairy milk and its plant‐based alternatives.

Choosing calcium‐fortified products is a common public health and nutrition professional recommendation regarding the selection of plant‐based products as dairy milk alternatives. However, while fortification can result in equivalent levels of calcium, it is important to consider that calcium, when added via fortification in plant‐based milk alternatives, has a lower bioavailability due to differences in type of calcium used for fortification, its solubility, sedimentation of fortified calcium, synergies with other nutrients that enhance its absorption in dairy milk, and the potential presence of inhibitors of bioavailability in plant‐based ingredients (Aydar et al., 2020; Heaney et al., 2000; Silva et al., 2020). For example, whole dairy milk holds calcium in suspension, but fortified calcium in alternatives may not remain dispersed, affecting utilization, intake, and bioavailability.

Research from New Zealand has shown that shaking plant‐based milk alternatives prior to use is necessary for calcium and other fortified nutrients to be transferred in the aqueous solution, otherwise it deposits on the bottom of the carton and is not consumed as intended, with decreased calcium levels of up to 97% for unshaken compared to shaken samples (Smith, Dave, et al., 2022). Shaking is also vital for the suspension of proteins in plant‐based milk alternatives, with large decreases seen in protein (by up to 66%) for unshaken samples, compared to those that were shaken before use (Smith, Dave, et al., 2022). Phytates are an example of inhibitors, often described as “anti‐nutrients” found in some plant‐based milk alternatives (including oat‐, soy‐, and cashew‐based milk alternatives) that may further decrease nutrient bioavailability via their impact on the absorption of nutrients including calcium, iron, zinc, and magnesium (Aydar et al., 2020; Eslami & Shidfar, 2019). Additionally, some polyphenols may inactivate thiamine or decrease the digestibility of proteins via interactions with digestive enzymes. However, it is not fully clear how processing impacts these interactions (Aydar et al., 2020).

A set of nutrient standards for plant‐based milk alternatives have been proposed in the United States to address the inconsistency in nutrient content, including minimum content requirements for protein, consistent fortification patterns, and maximum allowable levels of fat, added sugars, and sodium (Drewnowski, 2021b, 2022). The variability in the nutrient composition of plant‐based milk alternatives exists both between and within source plant categories. As such, some are broadly nutritionally similar to dairy milk, while others are substantially different (Table 1). Some plant‐based milk alternatives score more highly than dairy milk in metrics such as the Nutrient‐Rich Food Index (NRF), while others attract negative scores (Blumfield et al., 2021; Drewnowski, 2021a, 2022). Data on the contribution of plant‐based milk alternatives to nutrient intake and adequacy globally are lacking.

### 2.3 Bioactives

Dairy milks and plant‐based milk alternatives also do not have equivalent profiles of bioactive compounds, despite both being naturally derived. These bioactives may have direct and indirect health benefits through interactions with nutrients. For example, bioactive peptides in dairy milk may lower blood pressure, and components of the milk fat globule membrane may reduce the absorption and impact of saturated fats on health (Fekete, Veuthey, et al., 2016; FitzGerald & Meisel, 2000; Marcone et al., 2017; Table 2). The bioactives in plant‐based milk alternatives vary by plant type and processing. Generally, plant‐based milk alternatives contain oligosaccharides and prebiotics, which may have benefits for gut health (Zhang et al., 2020). Some oat milk contains beta‐glucans (depending on processing) which may assist with cholesterol management, and soy and chickpea beverages contain health‐promoting isoflavones (Siddiqui et al., 2023). However, the bioactive profiles of plant‐based milk alternatives remain understudied, and bioactive contents and subsequent health effects are often inferred based on the presence of the whole plant, not the processed aqueous solution that is used as milk. Importantly, the proportion of plant‐based ingredients in plant‐based milk alternatives is relatively low, ranging from 2% (nut and seed) to 20% (coconut) (Zhang et al., 2020), indicating low potential to provide plant‐based bioactives, especially when potential losses during processing are considered (Aydar et al., 2020; Tong et al., 2022).

**TABLE 2:** Major bioactives and synergistic components in dairy milk and plant‐based milk alternatives.

Component	Impact
*Dairy milk*	
Milk fat globule membrane (MFGM)	Stabilizes fat globules in solution, protects the fat globule from degradation, and likely plays a key role in regulating the digestion and absorption of milk fats (Lin et al., 2021; Weaver, 2021) Contains both bioactives and nutrients (including fat‐soluble vitamins, lipids, and proteins) (Feeney & McKinley, 2020; Weaver, 2021) The protein components of MFGM have demonstrated anticancer and antibacterial properties, and have a role in supporting immune function and lowering the risk of heart disease (Lin et al., 2021) The lipid components have bioactivity associated with maintaining gut function, supporting the immune system, and preventing the accumulation of cholesterol (Lin et al., 2021)
Bioactive peptides and proteins	Short strings of 2–20 amino acids are produced during the digestion of milk proteins, including both whey (such as lactoferrin) and casein proteins (Ahvanooei et al., 2022; Auestad & Layman, 2021) Shown to have a wide range of bioactivities including antihypertensive, antimicrobial, antithrombotic, immunomodulatory, antioxidative, antidiabetic, and mineral‐binding functions (Auestad & Layman, 2021) Whey proteins (alpha‐lactalbumin and beta‐lactoglobulin) and amino acids (L‐lysine and L‐arginine) bind to and slowly release calcium during digestion, enhancing absorption (Fishbein, 2004; Guéguen & Pointillart, 2000). Phosphopeptides: Produced during casein digestion, and protect calcium from precipitation in the intestine (Fishbein, 2004; Mykkänen & Wasserman, 1980) and may also enhance absorption of other minerals (Melse‐Boonstra, 2020a)
Lactose	May enhance the absorption of calcium by influencing the structure of the gut lining, however, this may be dependent on lactose dose and age (Cochet et al., 1983; Pansu et al., 1981)
*Plant‐based milk alternatives*	
Prebiotics	May have benefits for gut health (Zhang et al., 2020)
Beta‐glucans	May assist with cholesterol management (oat milk only, depending on processing) (Siddiqui et al., 2023)
Isoflavones	Anti‐inflammation, anti‐cancer, anti‐obesity, anti‐diabetes, gut biota regulation, and osteoporosis prevention (Siddiqui et al., 2023)

### 2.4 Processing and the milk matrix

Dairy milk is often represented as “unnatural,” and plant‐based milk alternatives are positioned as a natural source of plant‐based nutrition (Schiano et al., 2020). In fact, dairy milk is a minimally processed, whole food. Dairy milk naturally contains hormones, such as estrogens, not found in plant‐based milk alternatives, with concentrations low and relative to endogenous levels in humans (Snoj & Majdič, 2018). Plant‐based milk alternatives are assembled from the processing of several and varied ingredients, often including isolates, additives, and preservatives. Additives are added during processing to promote consumer acceptance via enhanced palatability, mouthfeel, and appearance, and include oils, salt, sugars, and gums, as well as nutrients for fortification purposes (Fructuoso et al., 2021; Silva et al., 2020). As such, the majority of plant‐based milk alternatives are classified as ultraprocessed foods in the NOVA classification system (Blumfield et al., 2021; Drewnowski, 2021b; Rodríguez‐Martín et al., 2023) while dairy milk is regarded as minimally processed (Monteiro et al., 2019).

While plant‐based milk alternatives are designed to mimic dairy milk in terms of use, it is not possible to mimic the natural and complex matrix of whole foods. The milk matrix represents not just the nutritional and bioactive contents, but how those contents interact in the formation of more complex structures, including maintaining the dispersion of calcium, protein, and other components in solution (Pingali et al., 2023; Sethi et al., 2016; Townsend et al., 2023). The milk matrix influences how the nutrients and bioactives within it are absorbed, and the overall activity and impact of those nutrients and bioactives within the body, which can differ when compared to these nutrients in isolation (Pingali et al., 2023; Sethi et al., 2016).

Most of the plant matrix from the seeds, nuts, or grains used to make plant‐based milk alternatives is lost, as ingredients are typically a small percentage of the product (i.e., 2%) or use isolated proteins from plants (i.e., 20% oat protein isolate) and due to high levels of processing. While nutrient matching through fortification may align some features of plant‐based milk alternatives with dairy milk, the importance of studying whole foods instead of single nutrients is becoming clear as potential interactions may affect the metabolic response to the whole food compared to its isolated nutrients (Jacobs & Tapsell, 2013).

## 3 EVIDENCE OF HEALTH BENEFITS

An extended history of long‐term and prevalent consumption means that there is a significant body of evidence surrounding the health impacts of dairy milk. A recent umbrella review uniting data from 41 meta‐analyses on 45 different health outcomes found that dairy milk consumption was more often related to health benefits than harms, with 200 mL intake per day associated with a dose–response pattern of reduced risk for multiple common conditions of public health concern (Zhang et al., 2021), including cardiovascular disease, stroke, hypertension, colorectal cancer, metabolic syndrome, obesity, and osteoporosis (Zhang et al., 2021). Additional beneficial associations have been identified for type 2 diabetes and Alzheimer's disease (Zhang et al., 2021). However, potential increases in risk were found for prostate cancer, Parkinson's disease, acne, and iron deficiency anemia in infancy (Zhang et al., 2021). Despite the conflicting outcomes between prostate and colorectal cancer, the Cancer Council of Australia recommends the consumption of dairy, including milk and calcium‐fortified plant‐based alternatives, in line with the Australian food‐based dietary guidelines (Council, 2023). Importantly, the paucity of evidence means there is no comparative umbrella review for the health effects of plant‐based milk alternatives.

Multiple health benefits have been linked to the calcium content in dairy milk (Flynn, 2003; Sorenson et al., 1988; Zhang et al., 2016), which is a partial motivation for calcium fortification in plant‐based milk alternatives. However, this represents a reductionist approach that neglects to consider the diversity of nutrients, bioactive compounds, and the interactions in the milk matrix provided by dairy milk (Townsend et al., 2023). For example, protein, bioactive peptides, and phosphorus, in addition to calcium, are also determinants of bone mass accrual and bone health supplied by dairy milk (Bu et al., 2021; Rizzoli, 2022). Furthermore, it appears that calcium from dairy sources, but not supplements, may positively influence fat metabolism to have an overall beneficial effect on cardiovascular health, with differing responses potentially due to interactions between calcium and other components in the dairy matrix (Bolland et al., 2010; Bu et al., 2021). This highlights the difficulties of designing plant‐based milk alternatives as culinary mimics when it comes to expecting matched health outcomes.

While it was previously thought that milk contributed to adverse health outcomes via the influence of the intake of saturated fats on low‐density lipoprotein cholesterol (LDL‐C) levels, randomized control trials have shown that milk proteins can significantly reduce blood pressure, cholesterol, and triacylglycerol levels (Fekete et al., 2015; Fekete, Giromini, et al., 2016), with no difference in LDL‐C levels following full‐fat versus skimmed milk intake (Engel et al., 2018). Similarly, low‐fat milk is often recommended based on lower energy levels compared to full‐fat milk, and fat content is a common reason for avoidance of dairy milk. However, the balance of evidence suggests neutral or beneficial relationships between dairy milk intake and obesity (Abreu et al., 2012; Babio et al., 2022; Barba et al., 2005; Clarke, 2019; Guo et al., 2018; Louie et al., 2011; Mirmiran et al., 2005; Vanderhout et al., 2016; Wang et al., 2016). This is likely due to satiety impacts and possible additional milk matrix interactions, as the milk fat globule membrane is known to regulate a variety of genes involved in lipid metabolism (Rosqvist et al., 2015). Again, this highlights the difficulties of designing alternatives with fully equivalent features.

Large‐scale production and consumption of plant‐based milk alternatives are relatively new, and the number of plants used to create products is varied. As such, the body of evidence related to their health effects is significantly smaller and often relies on inferred benefits based on research on the whole plant food raw ingredient prior to processing, rather than the processed milk alternative product. The paucity of evidence means there are not umbrella reviews or meta‐analyses to draw on for most. The notable exception is soy‐based milk alternatives, which show benefits for cardiovascular health and all‐cause mortality (Hassan Sohouli et al., 2021; National Health and Medical Research Council, 2013; Rafferty & Heaney, 2008; Zhou et al., 2023). Importantly, data for soy milk cannot be directly applied to other products. While plant‐based milk alternatives are often positioned as part of a “plant‐based” diet, the evidence regarding the benefits of plant‐based diets is based on the consumption of unprocessed and minimally processed plant foods rather than processed foods and beverages made from plants (Pingali et al., 2023). The initial plant ingredient may be high in beneficial bioactive phytonutrients, proteins, dietary fibers, fatty acids, and vitamins, but these exist in significantly smaller amounts following processing, with estimates of loss between 80% and 90% for plant‐based milk alternatives (Pingali et al., 2023). More research on the health effects of these products is needed, as this “health halo” effect may lead to consumers perceiving these products as more healthful than the evidence base supports (Curtain & Grafenauer, 2019; Pingali et al., 2023).

### 3.1 Allergies and intolerances

Plant‐based milk alternatives serve a particular subset of the population with allergies or intolerances to dairy milk. Dairy milk allergy is one of the most common food allergies in early life, with an estimated prevalence in developed countries ranging from 0.5% to 3% at 1 year of age (Kattan et al., 2011; Saito et al., 2019). Prevalence in adults is significantly lower, with estimates around 0.5% (Rona et al., 2007). In contrast, soy allergy rates are lower in children, at around 0.4% prevalence (Kattan et al., 2011), but the prevalence in adults is similar at approximately 0.7% (Taylor et al., 2021). Tree nut allergy has a prevalence of 1–3% (McWilliam et al., 2020). For most of the population, there is no need to avoid these allergens, and those with allergies are likely to need more modifications of eating patterns to ensure nutritional adequacy, and these modifications are harder to achieve on a population level, compared to an individual one.

Lactose intolerance is a commonly cited reason for dairy milk avoidance. However, there is significant ambiguity and misunderstanding surrounding the descriptions and dietary management strategies available, with binary approaches (excluding all dairy) often applied (Fassio et al., 2018; Misselwitz et al., 2019). Lactose malabsorption occurs due to reduced activity in the enzyme lactase that breaks down the naturally occurring sugar in dairy milk, lactose. When lactose is ingested, the non‐digested lactose passes through the gut without being absorbed in the brush border of the small bowel mucosa (most commonly due to lactase nonpersistence) (Misselwitz et al., 2019). This lactose can then undergo bacterial fermentation in the colon, increasing the osmotic load and resulting in symptoms (e.g., abdominal pain, bloating, flatulence, constipation, and diarrhea). When malabsorption is coupled with these symptoms, it is usually referred to as lactose intolerance (Misselwitz et al., 2019). This can lead to significant anxiety and, therefore, complete avoidance of dairy to alleviate symptoms (Fassio et al., 2018; Misselwitz et al., 2019).

It is estimated that ~70% of the world's population has limited lactase enzyme activity (Misselwitz et al., 2019). This does not mean that 70% of people are lactose intolerant, as there is considerable variability in the severity of clinical manifestation within and between individuals. This depends on the level of persisting enzyme activity, volume of lactose ingested, and other foods eaten, and some may experience little to no adverse symptoms (Fassio et al., 2018; Misselwitz et al., 2019). While individuals are often prescribed, or self‐select, a lactose‐free diet, avoidance of all dairy is no longer the gold‐standard recommendation as this can contribute to micronutrient deficiencies. Most patients can tolerate 5–12 g of lactose per single dose, equivalent to approximately 100–250 mL of dairy milk, and increased if the lactose is consumed together with other nutrients or spread through the day (Fassio et al., 2018; Misselwitz et al., 2019). A low‐lactose diet, incorporating lactose‐free products, and spacing dairy milk consumption throughout the day or with meals can be strategies to avoid the complete elimination of dairy milk and the associated challenges for nutrition and behavioral modifications (Facioni et al., 2020; Fassio et al., 2018; Misselwitz et al., 2019).

## 4 THE POSITION OF MILK AND PLANT‐BASED MILK ALTERNATIVES IN FOOD‐BASED DIETARY GUIDELINES

While a fundamental concept of food‐based dietary guidelines is to provide dietary guidance in a manner that is “food‐based,” and in the context of dietary patterns, many guidelines express the health value of foods in terms of their nutrient content only (Comerford et al., 2021). Dairy foods are listed as a core food group in approximately two‐thirds of food‐based dietary guidelines globally (Comerford et al., 2021).

Almost half of food‐based dietary guidelines address plant‐based alternatives for animal foods (milk and meat) (Comerford et al., 2021). Phrasing is often focused on specific plant‐milk alternatives (such as soy) and does not necessarily reflect the broad diversity of plant‐based milk alternatives available on the market. For example, the Australian (Australian Guide to Healthy Eating, 2013) and the US guidelines (2020–2025) both consider dairy milk a core food, with plant‐based milk alternatives considered alternatives if they are fortified with calcium, although soy milk is mentioned specifically. This framing may lead consumers to believe that foods within the same core food group are equivalent in terms of nutrients and health potential. Conversely, the Canadian dietary guidelines have moved away from dairy as a core food group and instead include dairy milk and soy milk in the core food group with high‐protein foods (Canadian Dietary Guidelines, 2019). Guidelines in Latin America and the Caribbean are diverse and several do not specify dairy milk needs but do include frequencies for consuming animal products (Comerford et al., 2021), meaning plant‐based and dairy milk would theoretically be subject to separate guidance. The diversity of positioning and recommendations globally is reflective of the contextual nature of guidelines and the ongoing variability and evolution of market offerings and scientific evidence surrounding them.

Food‐based dietary guidelines tend to focus on recommending reduced‐fat dairy milk options above full‐fat varieties. This emphasis is based on reducing overall energy intake to meet both energy and nutritional needs and reflects a need to update recommendations surrounding saturated fats to align with more contemporary evidence (Australian Guide to Healthy Eating, 2013). The Heart Foundation of Australia has recently released an updated position statement on dairy and heart‐healthy eating, advising that for the general population, regular fat dairy products can be enjoyed every day (Heart Foundation, 2023), which reflects data showing that saturated fat from dairy milk does not contribute to adverse health impacts in the same way as saturated fats from other animal food sources. Importantly, dairy milk can be included as part of established health‐promoting dietary patterns. Two to three servings per day can be incorporated into the Mediterranean diet and the dietary approaches to stop hypertension (DASH) diet (D'Alessandro et al., 2019; Miller et al., 2006).

Plant‐based alternatives meet the needs of specific populations, such as consumers avoiding lactose or reducing energy and fat intakes, vegans, or those with allergies. However, it is essential that there is awareness of the limitations of plant‐based milk alternatives when it comes to nutrition, and the lack of nutritional and bioactive equivalence to enable consumers to seek health advice and plan diets where shortfall nutrients and functional alternatives to bioactives can be obtained from other sources. When price is considered, dairy milk generally has a lower unit cost for most nutrients, with the exception of oat milk being a more affordable source of zinc, compared to dairy (Ramsing et al., 2023). This means that most plant‐based milk alternatives are less accessible to low‐socioeconomic groups (Sethi et al., 2016), who may be particularly vulnerable to deficiencies of the shortfall and priority nutrients provided by milk.

## 5 ENVIRONMENTAL IMPACTS

Making a more environmentally friendly choice is cited as a reason to choose plant‐based milk alternatives over dairy milk, as dairy milk production is considered resource intensive in terms of water and land use, and carbon footprint. However, these comparisons are typically made based on volume, without regard for nutritional value.

**FIGURE 1:**
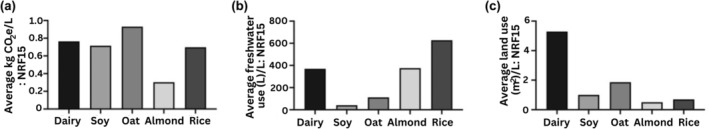
Contribution of dairy milk and plant‐based milk alternatives to CO_2_ equivalent emissions, freshwater, and land use, with respect to nutrient‐rich food score (NRF15). Data on environmental metrics from *Our World in Data* Accessed October (2023) ratioed to NRF15 scores calculated as per Sluik et al. (2015).

While dairy milk has a significantly higher contribution to CO_2_ equivalents (a unit of measurement that is used to standardize the climate effects of various greenhouse gases) compared to plant‐based milk alternatives on a per liter basis (*Our World in Data* Accessed October 2023), when nutrient density [e.g., using the NRF index NRF15 which factors in levels of protein, dietary fiber, monosaturated fats, vitamins A, B_1_, B_2_, B_12_ C, D, E, folate, calcium, iron, potassium, and zinc (Sluik et al., 2015)] is factored in, dairy milk, rice‐ and soy milk alternatives have a similar environmental contribution, and oat milk has a higher contribution (Figure 1a) (*Our World in Data* Accessed October 2023). Likewise, water use is significantly higher for dairy milk on a per liter basis, but relative to NRF15, almond and rice milk have higher requirements (Figure 1b) (*Our World in Data* Accessed October 2023). However, dairy remains highest in requirements for land use regardless of nutritional contribution (Figure 1c) (*Our World in Data* Accessed October 2023).

One review of what constitutes a sustainable and healthy diet, the EAT‐Lancet report, suggests that the optimal consumption of dairy foods includes a range of 0–500 g per day (Willett et al., 2019), with 500 g being equivalent to two serves of milk in the Australian Guide to Healthy Eating (which recommends 2.5 serves of dairy per day for adults aged 19–50 years) (NHMRC, 2013). This AGHE guideline was developed using dietary modeling to ensure that all nutrient requirements were met (Byron et al., 2011). In contrast, modeling to assess nutrient intakes under EAT‐Lancet recommendations has suggested that for adults and women of reproductive age, estimated intakes of vitamin B_12_, calcium, iron, and zinc were well below recommended intake levels (Beal et al., 2023). It is also important to note that the EAT‐Lancet report specifies that it does not imply that the global population should eat the exact same foods, nor does it prescribe an exact diet (Willett et al., 2019). Rather, local interpretation and adaptation are necessary and should reflect the culture, geography, and demography of the population and individuals (Willett et al., 2019). This means that there may be a gap between EAT‐Lancet recommendations and what is optimal for adequate nutrient intake and health in each country (Willett et al., 2019).

## 6 CONCLUSIONS

As plant‐based milk alternatives continue to grow in popularity, there is a risk that consumer beliefs, as well as assumptions underpinning food‐based dietary guidelines, may not align with the available evidence. The framing of plant‐based milk as “alternatives” to dairy milk is based on their intended use, and focuses only on specific nutrients, without considering other nutrients, synergy between nutrients, bioavailability, health effects, or other modifying factors such as the milk matrix. Presently, while plant‐based milks are framed as alternatives, they should not be perceived as equivalents due to the complex differences between types.

Consumers may choose plant‐based milk alternatives for a variety of reasons including dairy milk allergies, lactose intolerance, animal welfare, taste preferences, environmental concerns, vegan or vegetarian dietary patterns, acne management, or other health concerns. These needs demonstrate the value of plant‐based milk alternatives as a product category, providing options to those who cannot or choose not to consume dairy. However, it is important not to conflate individual needs with population‐level dietary guidance and health recommendations. The health halo applied to plant‐based milk alternatives, based on the properties of their raw ingredients, may mislead consumers regarding health benefits. This may have unintended consequences for the nutritional adequacy of populations and impact disease risks. Similarly, considerations of environmental attributes of plant‐based milk alternatives, without considering their nutritional density, may also skew decision‐making away from dairy milk at the expense of the consumption of key nutrients, depending on the plant‐based milk alternative chosen, either resulting in intake insufficiencies or requiring that these nutrients be obtained from elsewhere, with further environmental impacts.

More research is needed regarding the health benefits of plant‐based milk alternatives, the bioavailability of different nutrients and bioactive, and consumer and healthcare professional perception, and the consequences of naming and labeling are necessary to ensure that the policymakers, manufacturers, consumers, and other stakeholders are adequately informed regarding decision‐making surrounding choices for milk.

## AUTHOR CONTRIBUTIONS

**Emma L. Beckett:** Conceptualization (equal); data curation (equal); investigation (equal); methodology (equal); project administration (equal); writing – original draft (equal); writing – review and editing (equal). **Tim Cassettari:** Conceptualization (supporting); data curation (supporting); funding acquisition (supporting); methodology (supporting); project administration (supporting); writing – review and editing (equal). **Carlene Starck:** Conceptualization (equal); data curation (equal); methodology (equal); project administration (equal); writing – review and editing (equal). **Flávia Fayet‐Moore:** Conceptualization (equal); supervision (equal); writing – review and editing (equal).

## FUNDING INFORMATION

This study received funding from The a2 Milk Company Limited.

## CONFLICT OF INTEREST STATEMENT

All authors independently work for or collaborate with FOODiQ Global, which gains project funding from government, not‐for‐profits, professional, community, and industry organizations. All authors declare no conflicts of interest. The funding body, The a2 Milk Company Limited, provided general feedback on the broad study topic; however, it had no contribution to the final methodology, implementation, interpretation of results, or drafting of the manuscript.

## Data Availability

As this is a commentary piece, all data have been collected from the references provided, and data sharing privileges sit with the original authors of those works.
